# Adaptive behavior is guided by integrated representations of controlled and non-controlled information

**DOI:** 10.1101/2025.08.07.669231

**Published:** 2025-08-11

**Authors:** Bingfang Huang, Harrison Ritz, Jiefeng Jiang

**Affiliations:** 1 Department of Psychological and Brain Sciences, University of Iowa, Iowa City, IA 52242, USA.; 2 Cognitive Control Collaborative, University of Iowa, Iowa City, IA 52242, USA.; 3 Princeton Neuroscience Institute, Princeton University, Princeton, NJ 08540, USA.; 4 Iowa Neuroscience Institute, University of Iowa, Iowa City, IA 52242, USA.

**Keywords:** cognitive control, integrated task representation, decoding, representational similarity analysis, item-specific proportion congruency, adaptive behavior

## Abstract

Understanding how task knowledge is encoded neurally is crucial for uncovering the mechanisms underlying adaptive behavior. Here, we test the theory that all task information is integrated into a conjunctive task representation by investigating whether this representation simultaneously includes two types of associations that can guide behavior: stimulus-response (non-controlled) associations and stimulus-control (controlled) associations that inform how task focus should be adjusted to achieve goal-directed behavior. We extended the classic item-specific proportion congruency paradigm to dissociate the electroencephalographic (EEG) representations of controlled and non-controlled associations. Behavioral data replicated previous findings of association-driven adaptive behaviors. Decoding analyses of EEG data further showed that associations of controlled and non-controlled information were represented concurrently and differentially. Brain-behavioral analyses also showed that the strength of both associations was associated with faster responses. These findings support the idea that controlled and non-controlled associations are governed by an integrated task representation to guide adaptive behaviors simultaneously.

## Introduction

Cognitive control coordinates our thoughts and actions with internal goals ^1,2^ via the abilities of sustained task focus when faced with distractions (stability) and flexible adjustments between task sets based on varying situations (flexibility). Both stability and flexibility can be implemented in a proactive ^3,4^ or reactive ^5,6^ manner ^7–13^. For task focus indicating cognitive stability, conflict paradigms have been employed to explore how the brain focuses on task-relevant information while avoiding the distraction from potent task-irrelevant information ^14–16^. For example, in the Stroop task ^14^, participants are shown a color word and asked to respond to the ink color of the word while ignoring the meaning of the word. The ink color can be consistent (congruent trials) or inconsistent (incongruent trials) with the meaning of the word, resulting in a conflict effect measured by worse performance on incongruent trials than on congruent trials. Some models of cognitive control postulate that the conflict effect reflects the involvement of cognitive control, which suppresses the habitual but inappropriate response to the word and/or boosts the novel and goal-directed response to the ink color to resolve the conflict on incongruent trials ^3,17^.

A key question in the research on cognitive control is how the brain monitors control demands (i.e., how much control is needed). Recent research has demonstrated the importance of associative learning in guiding control demand ^18–21^. In particular, theories focusing on stimulus-control (SC) associations assume that the brain binds together a stimulus feature with the concurrent control demands ^6,19,22–32^. For example, in the Stroop task, if the color red is used on mostly congruent (MC) trials and the color blue is presented on mostly incongruent (MI) trials, then red would be associated with lower control demands relative to blue. This process has been proposed to account for the item-specific proportion congruency (ISPC) effect ^33,34^, whereby the conflict effect is reduced for stimulus features that predict higher control demands.

The ISPC effect is a natural prediction of stimulus-control learning, with the potential to provide a theoretical foundation for how cognitive control is scaffolded on structured representations of our environment. However, there have been theoretical challenges that the ISPC may arise from lower-level stimulus-response (SR) learning theory instead. This alternative account posits that associations may also form between stimulus and specific response in addition to more abstract SC associations ^35–38^. Returning to the Stroop task, if the word blue is often presented in red color, then the word blue will be associated with the ‘red’ response (in Stroop, often an assigned keypress or verbal response). Subsequent responses to this stimulus will be biased towards the associated response, whether or not they are correct.

Since SC and SR accounts often predict similar behavioral patterns, previous studies have typically focused on one at a time through experimental controls, and current empirical evidence supports both SC ^30,39–41^ and SR ^35^ associations. Additionally, studies of ISPC have differentiated the behavioral effects and neural substrates of SC and SR associations when either SC or SR associations are available ^23,29^. For example, Chiu and colleagues ^23^ showed that the dynamics of SC and SR learning were captured by trial-wise prediction errors with a reinforcement learning model and that SC learning was encoded in the caudate nucleus while SR association was represented in the parietal cortex. Conceptually, SC associations affect behavior by modulating other cognitive processes via cognitive control, whereas SR associations drive behavior in a non-controlled manner by retrieving the associated response. These accounts provide competing explanations for what kind of knowledge is encoded in neural task representations. Task representations are often thought to be conjunctive, providing one-point access to all task-related information ^5,42–51^. However, it remains unexplored whether and how controlled and non-controlled information can be integrated into a task representation to guide adaptive behavior. If controlled and non-controlled information are encoded in the same representation, then: (1) SC and SR representations should occur simultaneously; (2) the strength of SC and SR representations, being in the same task presentation, should covary (i.e., positively correlated) over time as the strength of the task representation fluctuates and (3) both SC and SR associations should exhibit behavioral relevance.

To test these predictions, we extended the classic ISPC paradigm to dissociate each of SC and SR associations from their associated stimuli (e.g., separating SC association from the stimulus and the linked control demand). Using this new ISPC paradigm in an EEG experiment, we tested whether SC and SR form integrated task representations using decoding, representational subspace analysis, and representational similarity analysis (RSA). We found strong evidence supporting task representations that integrate controlled (SC) and non-controlled (SR) associations. First, time-resolved RSA on the decoding results showed simultaneous representations of SC and SR associations. Second, the strength of SC and SR associations was positively correlated, consistent with the hypothesis that both SC and SR associations are integrated in a task representation. Third, both the strength of SC and SR associations were associated with faster responses at the trial level, indicating the behavioral relevance of the neural representations. These results provide convergent evidence of an integrated task representation that contains both SC and SR associations to jointly guide goal-directed behaviors.

## Results

### Task overview.

**Figure 1 |:**
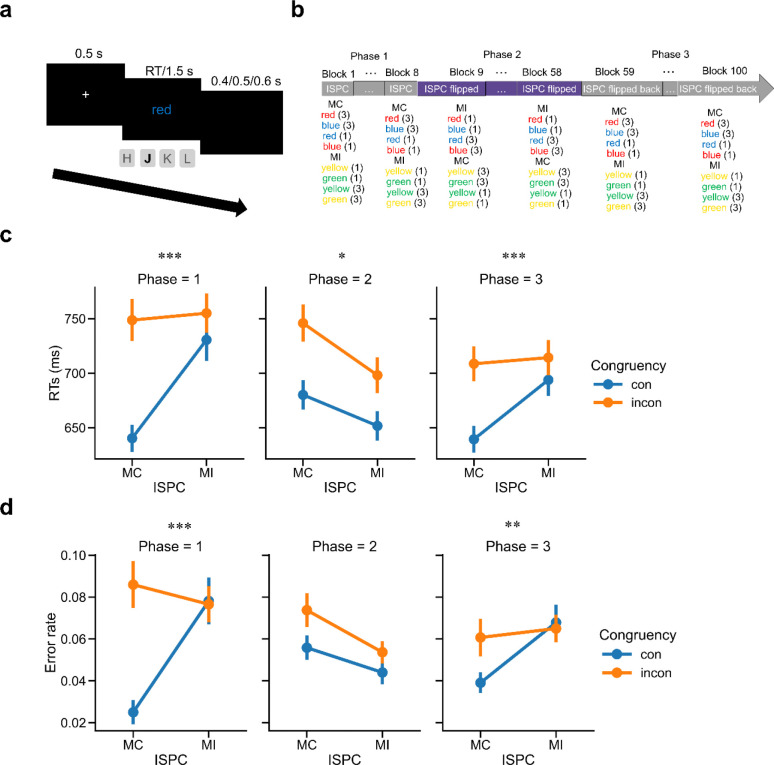
Experimental design and behavioral results (N = 40). (**a**) Trial structure. (**b**) Procedure of the main experiment. The number following each stimulus indicates its trial count within a mini-block. (**c**) Group mean RT as a function of the ISPC effect. (**d**) Group mean error rate as a function of the ISPC effect. Error bars show standard errors of the mean (SEM). MI: mostly incongruent trials; MC: mostly congruent trials; incon: incongruent trials; con: congruent trials. *: p < 0.05; **: p < 0.01; ***: p < 0.001.

EEG data were acquired while participants (N = 40) performed a 4-key Stroop task. On each trial, participants responded to the color of a word while ignoring the identity of the word (e.g., RED presented in blue color; Fig. 1a). Participants were encouraged to respond as quickly and accurately as possible. We applied three key manipulations to this experiment (Fig. 1b). First, each stimulus could be either congruent or incongruent, defined by whether the color was consistent with the word. Second, to test whether controlled and non-controlled information are encoded in a task representation, we manipulated the ISPC ^23,29,33,52^ in Phase 1 to allow the associations between colors and their paired cognitive control demands (stimulus-control; SC) and the associations between words and their linked responses (stimulus- response; SR) to be induced at the same time. To keep the overall proportion of congruency at 50%, one set of colors was presented on 75% of congruent trials (mostly congruent trials, or MC), whereas the other set of colors was presented on 25% of congruent trials (mostly incongruent trials, or MI). The MC manipulation pairs color with low control state based on SC assumption (e.g., red-congruent) and word with consistent response (e.g., red-red) based on SR account. Similarly, the MI manipulation links color to high control state (e.g., blue-incongruent) and word to inconsistent response (e.g., yellow-green, because each set has two colors, the inconsistent response is unique). This standard ISPC manipulation can test whether neural representations of controlled and non-controlled information are wrapped on the same trial by combing with the following EEG analysis (See method). However, it potentially mixes color identity with SC, word identity with SR, and the ISPC between SC and SR. To de-confound these factors and obtain pure estimates of SC and SR on each trial, we modified this paradigm by flipping the ISPC contingencies across different phases of the task. For example, the color ‘red’ might be associated with 25% congruency in Phase 1, 75% congruency in Phase 2, and then return to 25% congruency in Phase 3 (see Methods). Overall, these manipulations resulted in a 2 (Congruency: congruent vs. incongruent) × 2 (ISPC: MC vs. MI) × 3 (Phase 1 – 3) within-subject factorial design.

### Behavioral patterns track manipulations of varying ISPC.

RTs on correct trials within ±3 standard deviations (SD) of the mean and error rates were analyzed separately in each phase using a 2 (Congruency: congruent vs. incongruent) × 2 (ISPC: MC vs. MI) repeated-measures ANOVA. For RT data, there was a statistically significant interaction between Congruency and ISPC in each phase (Phase 1: *F*_(1,39)_ = 36.36, *p* < 0.001, *η*_ρ_^2^ = 0.482; Phase 2: *F*_(1,39)_ = 6.63, *p* = 0.014, *η*_ρ_^2^ = 0.145; Phase 3: *F*_(1,39)_ = 53.27, *p* < 0.001, *η*_ρ_^2^ = 0.577), which replicated the ISPC effect of smaller congruency effect in the MI condition compared with that in the MC condition in each phase (Phase 1: *t*_39_ = 6.03, *p* < 0.001, Cohen’s *d* = 1.174; Phase 2: *t*_39_ = 2.58, *p* = 0.014, Cohen’s *d* = 0.476; Phase 3: *t*_39_ = 7.30, *p* < 0.001, Cohen’s *d* = 1.317; Fig. 1c). For error rate, the 2-way interaction was significant in Phase 1 (*F*_(1,39)_ = 18.05, *p* < 0.001, *η*_ρ_^2^ = 0.316) and Phase 3 (*F*_(1,39)_ = 10.43, *p* < 0.01, *η*_ρ_^2^ = 0.211) and was driven by smaller conflict effects in the MI condition than in the MC condition (Phase 1: *t*_39_ = 4.25, *p* < 0.001, Cohen’s *d* = 0.842; Phase 3: *t*_39_ = 3.23, *p* < 0.01, Cohen’s *d* = 0.655; Fig. 1d), but was not significant in Phase 2 (*F*_(1,39)_ = 1.50, *p* > 0.05). The above findings showed that the ISPC effect flipped from Phase 1 to Phase 2, and then reversed again in Phase 3, indicating that the experimental manipulations worked as expected. Additionally, we replicated the congruency effect (i.e., worse performance on incongruent than congruent trials) in both RT and error rate data in each phase (Supplementary Note 1 and Supplementary Table 1).

### Decodable and separate representations of controlled and non-controlled associations.

**Figure 2 |:**
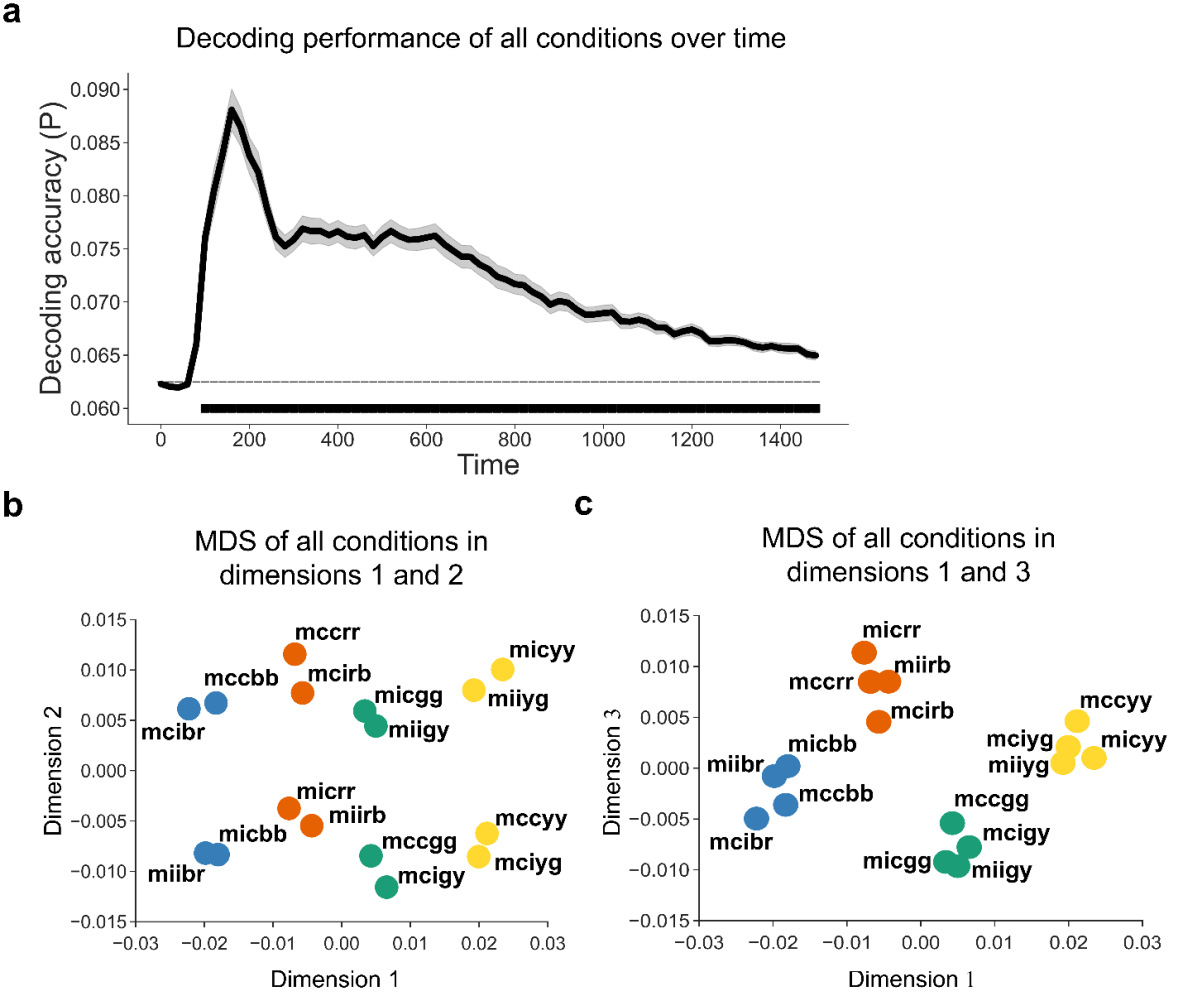
Decoding performance including both SC and SR latent subspaces (N = 40). (**a**) Group average decoding accuracy of all 16 experimental conditions as a function of time after stimulus onset. Shaded regions represent SEM. The solid line above the time axis denotes the time points showing above-chance (0.0625, or 1/16) decoding accuracy (p < 0.001, Bonferroni corrected). (**b, c**) MDS of EEG data across all experimental conditions. The label of each dot encodes the condition in the experimental design in the order of ISPC, congruency, color, and word. For example, “mccbr” means the condition with MC, congruent trial, blue color, and the word “red”. The color of each dot denotes the ink color of each condition.

Before exploring the relationship between controlled and non-controlled representations, we first confirmed that each representation was encoded during task performance. We applied linear discriminant analysis (LDA) ^53^ with the features of 64-channel event-related potentials (ERP) data to decode the 16 experimental conditions (8 unique stimuli × MC/MI) at each time point. The decoding analysis was performed on a time window from stimulus onset to 1500ms post-stimulus onset (cf., Fig. 1a). We found that the decoding accuracy was significantly above chance level from ~100ms after stimulus onset to the end of the analysis time window (Fig. 2a). To illustrate the distribution of data in the feature space, we applied multi-dimensional scaling (MDS, see Methods) to the ERP data averaged over 100 – 1500 ms after trial onset and visualized the centers of the 16 experimental conditions using the first 3 dimensions. The cumulative explained variance of the first 3 dimensions in the MDS was 0.61, 0.78 and 0.88, respectively. Dimensions 1 and 2 represented the 16 conditions in 8 SC classes (4 colors × MC/MI, Fig. 2b). MDS also categorized the conditions as 4 clusters denoting 4 colors along dimensions 1 and 3 (Fig. 2c).

**Figure 3 |:**
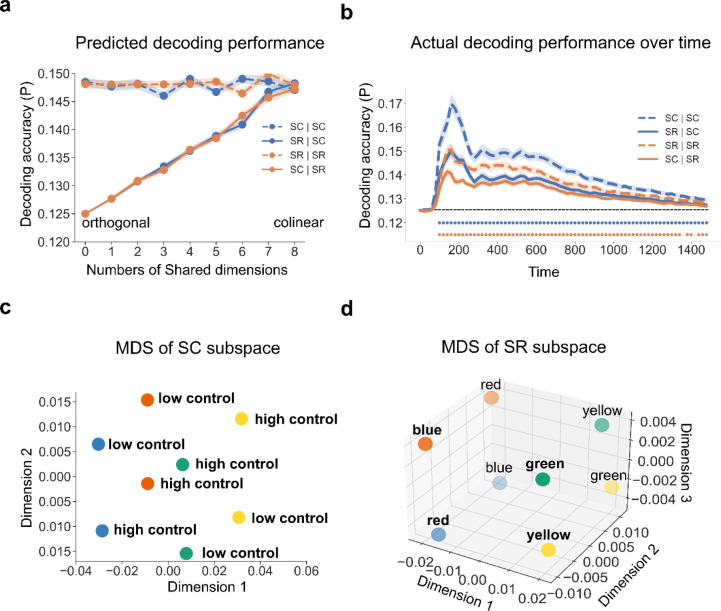
Partially overlapping SC and SR subspaces. (a) Simulation results (N = 40) of decoding accuracy as a function of the degree of subspace overlap, subspace type, and decoder. Both SC and SR subspaces are 8-dimensional (SC: 4 colors × MC/MI; SR: 4 words × 2 possible responses per word). The number of Shared dimensions indicates how many dimensions overlap between SC and SR subspaces. Each condition label is encoded in the format of “subspace | decoder”. For example, “SC | SR” means a SR decoder trained on the SC subspace. (b) Group average decoding accuracy over time as a function of which subspace the decoders are trained on. Shaded regions represent the SEM. Blue (orange) points denote the time points showing significantly better decoding accuracy when using the same subspace than when using the other subspace (p < 0.001, Bonferroni corrected). (**c**) MDS of the SC subspace. Each dot represents the center of a SC class. Dot color and label encode the ink color and cognitive control state, respectively. (**d**) MDS of the SR subspace. Each dot represents the center of a SR class. The label and dot color encode the word meaning and associated response, respectively.

Prior to testing whether controlled and non-controlled associations are represented simultaneously, we first tested whether the two representations are separable in the EEG data. To this end, we leveraged the subspace created by the LDA (see Methods). Briefly, to capture the subspace that best separates our experimental conditions, we projected our data onto the decoding weights of our LDA, forming 8-dimensional subspaces for SC (4 colors × MC/MI) and SR (4 words × 2 possible responses per word). We hypothesized that if SC and SR subspaces are identical, representations of SC and SR associations should not be separated. On the other hand, if SC and SR association representations are in different subspaces, the SC/SR subspace will not encode all information for SR/SC associations. As a result, decoding accuracy should be higher using its own subspace (e.g., decoding SC using the SC subspace) than using the other subspace (e.g., decoding SC using the SR subspace). We used cross-validation to avoid artificially higher decoding accuracy for decoders using their own subspace (see Methods). We verified this hypothesis in computational simulations to understand how the representational subspace analysis should perform under different accounts (see Methods). The simulation results (Fig. 3a) showed that: (1) when SC and SR subspaces are completely separable (i.e., the two subspaces shared no dimensions), decoding accuracy using the other subspace is at chance level; (2) when the SC and SR subspaces partially overlap (i.e., share some but not all dimensions), decoding accuracy using the other subspace is above chance level but less than when using its own subspace; and (3) when the SC and SR subspaces fully overlap (i.e., share all dimensions), decoding accuracy using the other subspace is above chance level and is comparable to that when using its own subspace.

In the EEG data (Fig. 3b), we observed that the decoders using the other subspace (i.e., SC | SR and SR | SC) showed significantly above-chance accuracy (p < 0.001, Bonferroni corrected) after ~100ms following stimulus onset. During the same time window, the decoder performance was significantly worse than the decoders using their own subspace (i.e., SC | SC and SR | SR). Thus, the empirical results are most consistent with the simulations of partially overlapped SC and SR spaces, suggesting that SC and SR association representations are separable in the EEG data. To visualize how data are represented in each subspace, we applied MDS to the SC (Fig. 3c, cumulative explained variance was 0.67 and 0.81 for the first 2 dimensions, respectively) and SR (Fig. 3d, cumulative explained variance was 0.75, 0.96, 0.99 for the first 3 dimensions) subspaces on EEG data averaged between 100 to 1500ms post-stimulus onset, which revealed the different structures of SC and SR encoded in the brain.

### Simultaneously represented and positively correlated controlled and non-controlled associations.

**Figure 4 |:**
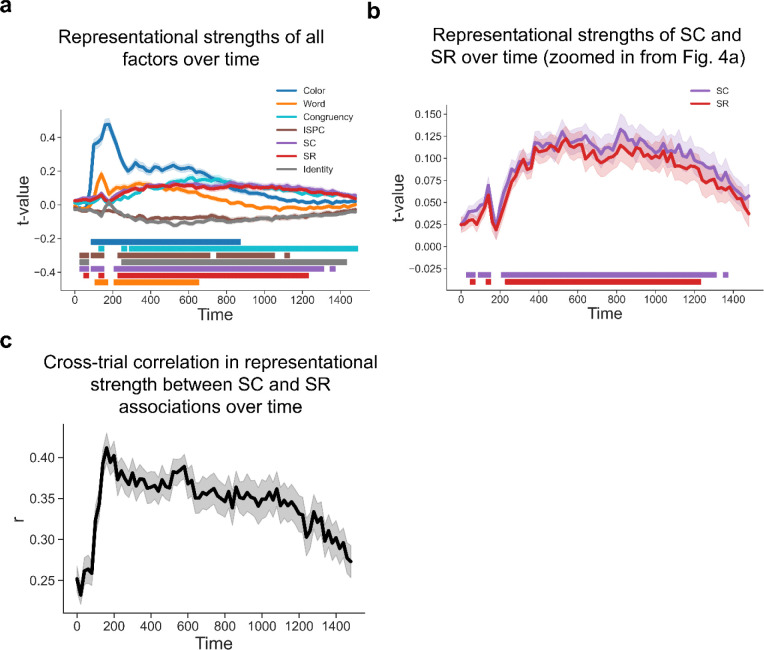
Simultaneous EEG representations of SC and SR associations. **(a**) Group average t values of representational strength for each factor over time. Squares below the lines indicate the significant time points (p < 0.001, Bonferroni corrected). (**b**) SC and SR association results from Fig. 5a. Shaded areas denote SEM. (**c**) Cross-trial correlation coefficient of representational strength between SC and SR associations, plotted as a function of time after stimulus onset. Shaded areas indicate SEM.

To further test whether SC and SR associations are simultaneously represented and contribute to adaptive task focus, we performed time-resolved RSA on decoding results to estimate the strength of representations of SC and SR associations while controlling for potential confounds (see Methods). In particular, since both SC and SR associations depend on the same ISPC condition, we included ISPC as a covariate in the same models as SC and SR associations to account for shared variability in the regression. The results showed that all the factors showed statistically significant effects on representational patterns during at least part of the trial time course (Fig. 4a). Crucially, both SC and SR effects remained statistically significant after ~200ms following the stimulus onset (Fig. 4a–b), suggesting that SC and SR associations were simultaneously represented.

The integrated task representation hypothesis predicted that the representational strength of SC and SR associations should be positively correlated across trials. On the other hand, it remained possible that at the trial level SC and SR were not represented simultaneously. That is, a trial might only show an SR effect but no SC effect, or vice versa. If this were true, SC and SR effects should be negatively correlated across trials. We observed a statistically significant positive correlation over all time points (Fig. 4c), indicating that trials showing a strong SC effect tended to show stronger SR effects as well. This finding further supports the hypothesis of simultaneously encoded SC and SR associations within an integrated task representation.

### Controlled and non-controlled associations jointly predict behavioral performance.

**Figure 5 |:**
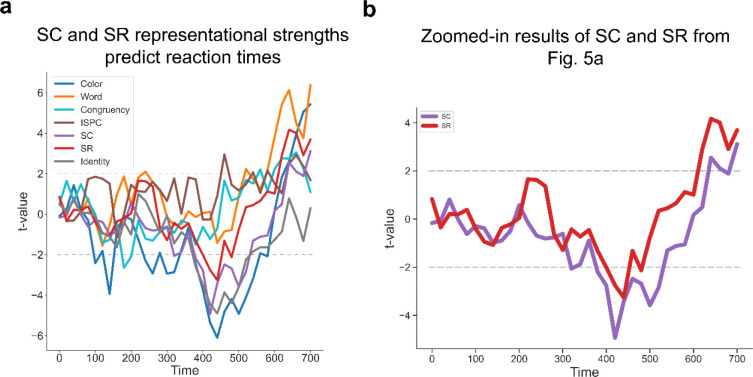
Both the strengths of SC and SR associations are correlated with RT. (**a**) Group averaged t-values for each factor predicting RT in the LMM analysis. (**b**) SC and SR association results from Fig. 5a.

To test whether the SC and SR effects from the RSA exhibited behavioral relevance, we predicted RT using a linear mixed model (LMM, see Methods) that included the representational strength of SC and SR associations, as quantified by the t-statistic from a trial-wise RSA. This analysis was conducted at each time point (time-locked to stimulus onset) prior to the group average RT (700.56ms) to test the effect of SC and SR representations on behavioral guidance. We found that faster responses were correlated with stronger representations of SC in the time window 380 – 520 ms (peak time: b = −0.009, SE = 0.002, *t*_56581.22_ = −4.93, *p=* 0.823^−6^) and SR in the time window 400 – 480 ms (peak time: b = −0.006, SE = 0.002, *t*_56580.80_ = −3.26, *p=* 0.001, Fig. 5a–b). These results suggest that controlled and non-controlled associations jointly influence performance before decision-making.

## Discussion

Adaptive behavior can be guided by associations for controlled processing (e.g., stimulus-control associations) and non-controlled processing (e.g., stimulus-response associations). To explore the relationship between these representations, we combined (1) a novel extension of a classic assay of associative learning in cognitive control with (2) neural subspace analyses of the time-resolved task representations. We found consistent support for distinct neural representations of SR and SC associations, but these associations appeared to share a synchronized temporal profile consistent with them constituting a common task representation.

We found behavioral ISPC effect in each phase of our task, even after the ISPC manipulations were flipped. This suggests that participants adjusted to the local ISPC conditions, which critically allowed us to dissociate stimulus features from their SC/SR associations. The behavioral ISPC pattern was consistent with previous studies ^23–26,28–30,33^. However, as both SC and SR associations are characterized by the same pattern of the ISPC effect, behavioral analysis and conventional contrasts between experimental conditions are insufficient to dissociate SC from SR effects. We addressed this issue with decoding, representational subspace analysis, and RSA on decoding results.

We also found that SC and SR associations were encoded in distinct representations but appeared to be temporally synchronized as a task representation. An SR association itself is automatic behavior, but it is sometimes included as a core component of cognitive control ^47^. The original motivation for the ISPC task, and the SC theory, was to explore the role of associative learning in cognitive control beyond automatic S-R relationships ^29,35,54^. Brain-behavior analysis prior to the mean RT supported the prediction that SC and SR can jointly guide goal-directed behaviors. Previous fMRI studies with typical ISPC paradigms showed that the ISPC effect was related to cognitive control area including the dorsolateral prefrontal cortex (dlPFC), the dorsal anterior cingulate cortex (dACC) and the parietal cortex ^55,56^. Therefore, the results showing that SC and SR had distinct subspaces but emerged in the same time window should not suggest a paradox of findings or functional equivalence of SC and SR; rather, they highlight the brain’s ability to encode controlled and non-control information in parallel with partially overlapping representational subspaces to serve goal-directed behaviors. Additionally, the caudate nucleus associated with SC and the parietal cortex related with SR were observed in separate SC or SR conditions ^23^. It is possible that SC and SR are supported by partially overlapping neural substrates with similar temporal dynamics. Direct evidence is needed to validate the possibility.

We further explored how controlled and non-controlled associations jointly affect performance via brain-behavioral analyses with LMM. In general, we observed that the strength of SC and SR association representations are both negatively correlated with trial-level variability of RT. There could be one alternative explanation that either SC or SR, instead of both, occurs on each trial. If this were true, the coefficients of SC and SR from RSA results should be negatively correlated across trials (i.e., some trials show strong SC effects and weak SR effects, while other trials show the opposite). However, we found the opposite pattern, which indicates that there is no competition between SC and SR to be represented. One possibility leading to this positive correlation is that both associations are encapsulated in an integrated task representation ^43,44,57^. Specifically, if the task representation is strongly activated on a trial, both SC and SR associations will also be strongly triggered, leading to a positive correlation between SC and SR effects across trials.

To interpret the ISPC effect, Bugg ^58^ proposed the dual item-specific mechanisms whereby SC and SR associations learning dominate the ISPC effect under different situations, which was supported by behavioral and imaging evidence ^23,29,30^. This means that SC associations would take over the adaptive effect when the frequency of incongruent trials is manipulated on task-relevant dimension, but SR associations would work when it is manipulated on task-irrelevant dimension. The results of the current study provide new temporal evidence for both control-driven ISPC and SR-driven ISPC and suggest that they can be represented simultaneously. Zooming in on the area of adaptive cognitive control, it has been debated whether adaptive task focus is actually involved with control or not, extending from ISPC to context/list-level PC ^40,59–77^ and the congruency sequence effect ^78–84^. Researchers have gradually formed an associative learning view on adaptive task focus ^10,12,13^. Egner ^12^ assumed integrating different levels of abstraction about associations including SR and SC into the same learning scheme serves for adaptive behaviors. The current study supports this assumption directly with the finding of joint action of control and non-control within the same stimulus for adaptive behaviors.

To better understand the role of complex associative learning in cognitive control, we developed a new way to test how representations are integrated, and how multiple representations of task information collaborate to guide goal-directed behaviors^47,85^. Previous research has employed partially overlapping tasks to test the conjunctive nature. For example, when there are two tasks producing different responses to the same input stimulus, encountering the stimulus will retrieve both task representations, leading to interference between the incompatible responses and impaired performance ^86–89^. However, this test is confounded by the retrieval of episodic memory (e.g., the last experiences performing the tasks, rather than the task representations), which is also conjunctive ^43,44,90^. Additional evidence supporting conjunctive task representation comes from studies showing that task identity is still decodable from electroencephalographic (EEG) data when accounting for individual task features such as stimulus, rule, and response ^48–51,91^. This approach relies on shared representations of task features across multiple tasks. It remained understudied how multiple task components within the same task are influenced by the conjunctive task representation. In this study, we extend the test of conjunctive task representation in two ways. First, we examine stochastic task features that must be learned from multiple episodes of task execution to tease apart retrieval of task representation from episodic retrieval. Second, we test the prediction that conjunctive task representation will simultaneously encode multiple task features instead of locating task-unique representations.

Despite the theoretical differences between SR and SC associations, our subspace analysis revealed a pattern that is consistent with partially overlapping subspaces for SC and SR representations. SC and SR are different in their concepts and information processing, but they shared variance in their operational definitions by both depending on the same ISPC conditions. While this may have resulted in the partially overlapping representations, it may also reflect simultaneous retrieval to guide behavior. Critically, our RSA analyses revealed concurrent representations of SC and SR associations even when controlling for ISPC confounds. The experimental design combined with the RSA method ensures that the predictors for SC and SR associations are different.

In summary, we find evidence of neural representations of associations of both controlled and non-controlled information quantified by SC and SR. The neural representations are represented simultaneously in an integrated representation in partially overlapping neural subspaces shortly after stimulus onset and remain active after the response is made. Moreover, the strengths of the associations prior to the response can jointly predict performance. These results shed light on how an integrated task representation, which consists of multiple associations that can drive behavior, can retrieve multiple associations of controlled and non-controlled information to guide adaptive behaviors.

## Methods

### Participants.

Sixty-one healthy adults participated in the EEG experiment. Sixteen participants with excessive muscle artifacts (6 subjects), too many corrupted EEG channels (5 subjects), and missed partial data (5 subjects), and five participants with accuracy below 75% were removed, resulting in a final sample of 40 participants (28 females, 12 males; age mean = 23.9, SD = 6.2, age information was not acquired for the first 25 participants). All participants had normal or corrected-to-normal vision. All participants gave informed consent prior to the experiment, and either were paid or received course credits for participation. All procedures were approved by the University of Iowa Institutional Review Board (UIIRB #202001345).

#### Stimuli.

The experiment was programmed using PsychoPy (ver. 2022.2.5). The stimulus was a color word presented in the center of the screen (24 inch LCD display with resolution of 1920×1080) on a black background. Four color words (red, blue, yellow, and green) and their corresponding colors were used. For each participant, the colors and their corresponding words were divided into two sets of red-blue and yellow-green. Within each set, the words and colors formed a 2 × 2 factorial design, resulting in eight unique Stroop stimuli ^14^ in total (e.g., red in red or blue color, blue in red or blue color, yellow in yellow or green color and green in yellow or green color).

### Design and procedures.

Each trial started with the presentation of a fixation cross for 500 ms, followed by a color word at the center of the screen (specified in ‘height’ with 0.08 units where the full screen height equaled 1.0 unit) for 1500 ms or until a response was made (Fig. 1a). The four colors were mapped onto four keys (“H” for red, “J” for blue, “K” for yellow and “L” for green). Trials were separated by a black screen for the remainder of the 1500 ms response deadline plus a jittered (400/500/600 ms with equal probability) interval. The trials were grouped into mini-blocks of 16 trials each. The participants first underwent a practice session with 3 mini-blocks of 50% congruent trials. During practice, participants received feedback for a correct, incorrect or too slow response on each trial. After reaching the 75% accuracy criteria across all blocks, they moved on to the main experiment (Fig. 1b) with 8 mini-blocks for phase 1, 50 mini-blocks for phase 2 and 48 mini-blocks for phase 3.

### Behavioral analysis.

Behavioral data from the main experiment were analyzed. For response time (RT) data, only correct trials within ±3 SD from each subject’s mean RT were included in the analysis. Accuracy and RT were analyzed separately for each phase using a 2 (congruency) × 2 (ISPC) repeated-measures ANOVA.

### EEG data acquisition and preprocessing.

EEG data were recorded from a 64-channel active electrode cap with the ground at Fz and the reference at Pz using an actiCHamp amplifier (Brain Products) at a sampling rate of 500Hz with a hardware band-pass filter from 0.016 Hz to 1000 Hz. All electrode impedances werekept below 10 kΩ during the experiment. All EEG data were analyzed using the EEGLAB toolbox (https://sccn.ucsd.edu/eeglab/index.php) and custom MATLAB scripts. Preprocessing started with rejection and interpolation of noisy electrodes. Next, a 0.1–50 Hz band-pass filter was applied to the raw EEG data. The data were then re-referenced to the average of all electrodes and segmented into epochs spanning from −500 ms to 1500 ms relative to the stimulus onset. We used independent component analysis (ICA) to remove eye-blink and muscle artifacts from the epochs. These epochs were baseline-corrected using a 200 ms window before stimulus onset. Epochs with amplitudes exceeding ± 80 μV were also rejected. Fewer than 70% of all the epochs were excluded for each subject.

### Decoding analyses.

**Figure 6 |:**
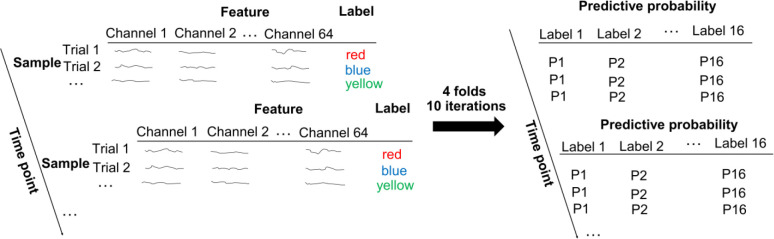
Illustration of linear discriminatory analysis (LDA).

To generate the dependent variable for the RSA, we first decoded the representations of all conditions using LDA (scikit-learn version 1.2.2 in Python ^92^). The preprocessed 64-channel ERP data were down-sampled to 50 Hz and were used as the features to decode the 16 experimental conditions (8 unique stimuli × MC/MI. Note that Phases1 and 3 shared the same ISPC manipulations). Decoding results were obtained by performing a 4-fold cross-validation procedure (Fig. 6). To avoid the bias of unbalanced trial numbers among different classes on decoding results, the more frequent trial types were down-sampled to be consistent with the less frequent trial types. Specifically, given the 75%/25% proportion congruency used in the manipulation of ISPC, the more frequent trial types had three times as many trials as the less frequent ones. Thus, the more frequent trial types were randomly divided into three parts, and one part was used for the 4-fold cross-validation. The cross-validation was repeated 30 times (i.e., 10 random partitions of the frequent trial types and each part being used once within each random partition). The LDA decoding accuracy was averaged. The decoding analysis was performed at each time point of a trial for each participant.

To construct SC and SR subspaces, we trained 8-way SC (4 colors × MC/MI) and SR (4 words × 2 possible responses per word) LDA decoders and then transformed all training and test data into the subspaces of the LDA decoders. For each subspace, we built and tested SC and SR decoders using the same cross-validation approach as above. To avoid bias, all decoders used the same partition of training and test data, with the class label of each trial determined by its experimental condition. To test the statistical significance of decoding results, Bonferroni corrections with a family-wise error rate of α = 0.001 were applied to address the issue of multiple comparisons. Additionally, classical multidimensional scaling (MDS) analysis ^93^ on a 16×16 matrix of decoding accuracy from the 16-way decoders was applied to visualize the neural representations of all conditions potentially including both SC and SR associations. Similar MDS analyses were performed on the 8×8 matrix of decoding accuracy from the SC decoder and the other 8×8 matrix of decoding accuracy from the SR decoder to visualize their neural representations.

### Simulations of cross-decoding accuracy.

To simulate SC and SR subspaces with different levels of overlap, we first constructed non-overlapping SC and SR subspaces, each having 8 dimensions and consisting of 8 classes of 100 data points each. The centers of the classes were equidistant, with the distance set to the mean between-center distance in the EEG subspace. For each class, the data points were randomly drawn from a multivariate Gaussian distribution using the class center as the mean and the within-class standard deviation from the EEG subspace (averaged across all time points). Both SC and SR decoding were then conducted on each of the SC and SR subspaces using 4-fold cross-validation. To examine how overlap in subspaces affects decoding accuracy, the overlap between the two subspaces was manipulated by the number of shared dimensions. That is, if n (n ≤ 8) dimensions were shared, n dimensions of data were randomly chosen for SC_SR (i.e., SR decoder trained on the SC subspace) and SR_SC decoders. For SC_SC and SR_SR decoders, all 8 dimensions were used regardless of n.

### RSA on decoding results.

**Figure 7 |:**
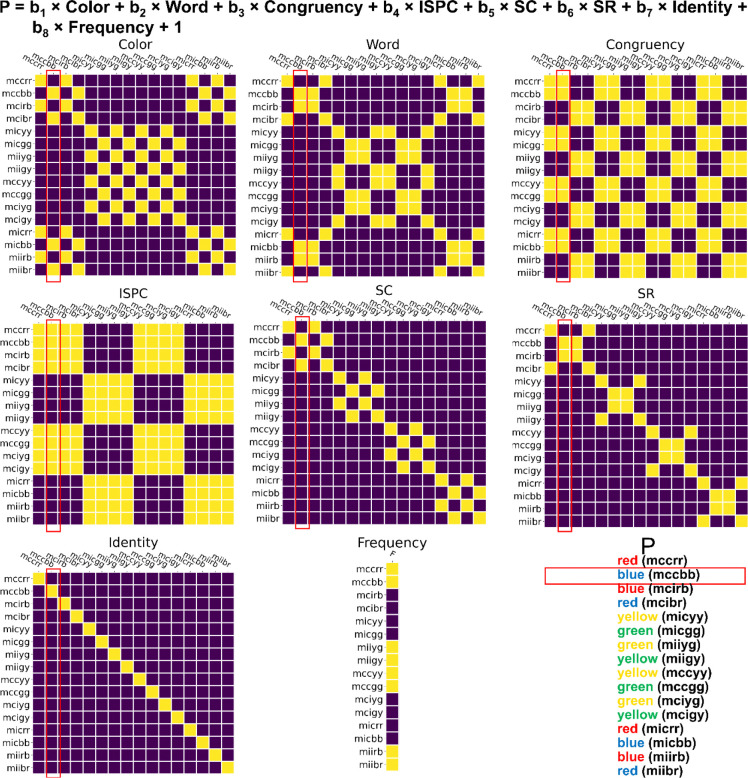
Illustration of RSA. The label of each row/column represents the condition in the experimental design including ISPC, congruency, color and word. For example, “mccbb” means the condition with MC, congruent trial, color blue and word blue. For each cell in a matrix, the color indicates whether the row and column conditions share the same factor (yellow = yes, blue = no) encoded by the matrix. For example, the cell at the 4^th^ row and the 2^nd^ column in the “SC” matrix encodes that the 4^th^ condition (i.e., mcibr) and the 2^nd^ condition (i.e., mccbb) share the same SC association.

To further track the representational dynamics of SC and SR associations, we tested the hypothetical representational similarity patterns using the empirical representational similarity patterns from EEG data and linear regression-based RSA ^48,49,91^. For each time point, its decoding results reflected the neural similarity between conditions (i.e., if two conditions have similar neural representations, the misclassification rate would increase) and were organized as the logit-transformed probability of a trial belonging to each of the 16 conditions to be the dependent variable. Six factors were considered, namely color, word, congruency, ISPC (MC/MI), SC (i.e., the pairing between color and ISPC), and SR (i.e., the pairing between word and ISPC), resulting in six hypothetical representational similarity patterns in the form of binary linear regressors (Fig. 7). Specifically, for each trial, each regressor was a column-vector of 16 cells, with each cell containing a binary value indicating whether each of the 16 conditions shared the feature (e.g., color) with the condition to which the current trial belonged to. Additionally, two nuisance binary regressors marking the condition and its frequency (i.e., whether a MC color on a congruent trial or a MI color on an incongruent trial) of the current trial were included. For each participant, the within-trial regressors formed a design matrix to regress against the empirical decoding results, resulting in trial-wise *t* values for each regressor. For each time point, individual mean *t* values for each regressor were submitted to a group-level one-sample *t*-test against 0. To test the significance of temporal representational strength, Bonferroni corrections with a family-wise error rate of α = 0.001 were applied to address the issue of multiple comparisons. Additionally, we correlated those regression coefficients of SC with that of SR across trials at each time point to test the possibility that SC and SR were not simultaneously represented within a trial.

### Linear mixed model (LMM).

We further performed LMM at each time point preceding the mean RT across all trials (700.56 ms) to test whether trial-wise RSA results could predict the RT variability ^48,91^. The model predicted trial-wise RT using fixed-effect predictors with *t*-values from the RSA regressors described above, plus random intercepts at the subject level. Log-transformed RTs were prewhitened by removing linear trends across trials and mini-blocks and then used as the dependent variable.
